# Environmental Dissemination of Multidrug‐Resistant Gram‐Negative Pathogens in Djibouti's Wastewaters

**DOI:** 10.1002/puh2.70139

**Published:** 2025-10-04

**Authors:** Ayan Ali Ragueh, Ibrahim S. Abdallah, Rachid M. Mouhoumed, Mohamed H. Aboubaker, Seydina M. Diene

**Affiliations:** ^1^ Université de Djibouti Campus Balbala Croisement RN2‐RN5 Balbala Djibouti; ^2^ Aix Marseille Univ, APHM, MEPHI, Faculté de Pharmacie, IHU‐Méditerranée Infection Marseille France; ^3^ Laboratoire de Biologie Médicale Mer‐Rouge Rue de Moscou Djibouti

**Keywords:** carbapenem resistance, Djibouti, extended‐spectrum β‐lactamase (ESBL), gram‐negative bacilli, wastewater

## Abstract

**Background:**

The occurrence of multidrug‐resistant (MDR) Enterobacteriaceae‐producing extended‐spectrum β‐lactamases (ESBLs) and/or carbapenemases is increasingly reported in clinical and environmental settings. Here, we aimed to investigate the resistance profile of gram‐negative bacterial isolates from urban and environmental wastewater in Djibouti.

**Materials and Methods:**

Twenty‐five wastewater samples were collected from eight different locations of Djibouti. After bacterial culture on selective media for gram‐negative bacteria (GNB), obtained isolates were identified using matrix‐assisted laser desorption/ionization time‐of‐flight (MALDI‐TOF) mass spectrometry. Antibiotic susceptibility testing was performed by disk diffusion and *E*‐test methods. Standard PCR and Sanger sequencing were used to investigate antibiotic resistance genes, including β‐lactamases, carbapenemases, and colistin resistance genes.

**Results:**

Eleven MDR gram‐negative isolates were identified from the 25 water samples collected, including six *Escherichia coli*, one *Enterobacter cloacae*, and four *Klebsiella pneumoniae* isolates. Interestingly, the pandemic ESBL gene *bla*_CTX‐M‐15_ was detected in 9 of the 11 (81.8%) from both urban and environmental water samples. In addition, the carbapenemase gene *bla*_OXA‐48_ was identified in 4 of the 11 strains (36.4%), including two *E. coli*, *one K. pneumoniae*, and one *E. cloacae*, all from urban wastewater, particularly hospital water samples.

**Conclusion:**

For the first time, we describe here MDR‐GNB bacterial isolates from urban and environmental wastewater in Djibouti. To date, carbapenem‐resistant isolates have only been present in hospital wastewater. Regular surveillance policies, consistent with the “One Health” approach, are recommended to prevent the spread and monitor the evolution of antibiotic resistance in the environment and within the Djiboutian community.

## Graphical abstract

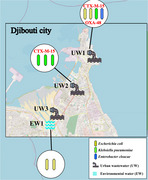

## 1 Introduction

Antibiotic consumption has become a critical aspect of modern medicine, significantly impacting both human and veterinary health. Antibiotics are essential in treating bacterial infections, preventing disease spread, and improving survival rates in both humans and other animals [1, 2]. However, their inappropriate use and overuse have significantly increased antimicrobial resistance (AMR). AMR poses an immediate threat to public health worldwide and was responsible for at least 1.27 million deaths globally in 2019, contributing to nearly 5 million deaths in total [3]. The discharge of untreated or minimally treated wastewater into the environment is a global issue that promotes the spread of antimicrobial‐resistant bacteria and resistance genes (ARG), presenting a major public health challenge [4, 5]. Moreover, in low‐ and middle‐income countries (LMICs), widespread antibiotic misuse, poor drug quality, inadequate sanitation infrastructures, and the concurrent release of untreated or poorly treated hospital, domestic, and agricultural wastewater into the environment significantly contribute to the prevalence of AMR in human infections [3, 4]. This situation is complicated by underdeveloped infectious disease surveillance programs in these countries, particularly regarding AMR [6]. Previous studies have demonstrated the role of wastewater, especially from healthcare facilities, as important reservoirs of AMR and carriers of multidrug‐resistant (MDR) bacteria released into the environment [7, 8, 9, 10]. Hospital wastewater is considered a key reservoir of antimicrobial‐resistant bacteria where horizontal gene transfers can occur between pathogens and commensals [11, 12]. In addition, urbanization has also significantly increased the presence of pathogenic bacteria and ARGs in aquatic environments [13]. The main threat of AMR is caused by gram‐negative bacteria (GNB), including Enterobacteriaceae, which are widely found in the environment due to their propensity to acquire antibiotic resistance. GNB infections include many types of infections in humans and animals [14, 15] and are particularly important in hospitals. β‐Lactam antibiotics are the most widely used drugs worldwide for treating bacterial infections in both humans and animals. Within this group, carbapenems are considered one of the last‐resort antibiotics in healthcare settings, and carbapenem‐resistant Enterobacteriaceae (CRE) can thus cause some of the most severe antibiotic‐resistant infections [16, 17]. NDM, KPC, and OXA‐48‐like enzyme expression by GNB has become the major mechanism of carbapenem resistance, and these enzymes are widespread in many parts of the world [18]. In Djibouti, there are generally few studies on MDR bacteria. Recently, studies have begun to examine the prevalence of MDR bacteria in human samples. However, no studies have reported the presence of AMR genes in the country's aquatic ecosystems. More importantly, no studies have examined the role of untreated wastewater as a source of contamination by resistant bacteria in the human population of Djibouti. The main objective of this study was therefore to investigate the presence of extended‐spectrum β‐lactamase (ESBL) and carbapenemase genes in GNB isolated from untreated wastewater in Djibouti and then to assess the potential contribution of these genes to the emergence of resistant bacteria.

## 2 Materials and Methods

### 2.1 Sample Collection

Between 20 and 28 February 2020, we collected environmental samples from several sites in the Republic of Djibouti, including untreated urban wastewater (UW) from areas near human habitations in the capital city (which included hospital wastewater) and environmental water (EW) from the main river valleys, or wadis, that cross the country. We collected the samples in sterile vials and immediately transferred them to the laboratory for storage at −80°C until further use. We aliquoted the collected water samples and incubated them in Tryptone Soy Broth (TSB, bioMérieux, Marcy l'Etoile, France) at 37°C for 72 h for enrichment.

### 2.2 DNA Extraction

We extracted genomic DNA from the enriched wastewater samples (after overnight incubation in TSB tubes at 56°C) using an EZ1 DNeasy Blood and Tissue Kit (Qiagen, GmbH, Hilden, Germany) according to the manufacturer's protocol. From extracted DNA, we conduct quantitative PCR (qPCR) to detect the presence of specific ARGs. The primers used targeted the following genes: ESBL genes (*bla*_TEM_, *bla*_SHV_, *bla*_CTX‐A_, and *bla*_CTX‐B_), carbapenemase genes (*bla*_OXA‐23_, *bla*_OXA‐58_, *bla*_OXA‐24_, *bla*_OXA‐48_, *bla*_NDM_, *bla*_KPC_, and *bla*_VIM_), and colistin resistance genes (*mcr*‐1 to *mcr*‐5 and *mcr*‐8). The primer sequences are presented in Table S1.

### 2.3 Bacterial Culture

For primary screening of resistant bacteria, we inoculated 20 µL from each TSB tube into MacConkey agar supplemented with ertapenem (0.5 µg/mL) and MacConkey agar supplemented with cefotaxime (1 µg/mL), followed by incubation at 37°C for 24 h. To prevent false‐negative cultures, we intentionally spiked portions of the collected samples with resistant strains to serve as positive controls. Bacterial colonies that grew on these two media were replicated on Columbia agar + 5% sheep blood (BioMérieux) for further analyses. Then identification of bacterial colonies from these media was done using the matrix‐assisted laser desorption ionization–time of flight (MALDI‐TOF) mass spectrometry (Bruker Daltonik, Bremen, Germany) [19].

### 2.4 Antimicrobial Susceptibility Testing and Molecular Analysis

The bacterial susceptibility to antibiotics was conducted on the Mueller–Hinton agar using the disk diffusion method (Fluka, St. Louis, USA) and interpreted according to EUCAST recommendations (EUCAST: www.eucast.org). The ESBL profile was detected through the presence of a “champagne cork” synergy between third‐ or fourth‐generation cephalosporins and clavulanic acid. The *E*‐test method (bioMérieux, Marcy‐l'Etoile, France) was used to determine the minimum inhibitory concentrations (MICs) of ertapenem and imipenem. Screening for carbapenemase activity was performed using the β‐CARBA assay (Biorad, Hercules, CA, USA). Finally, we performed standard PCR using positive and negative controls to confirm the presence of ARGs on the bacterial strains analyzed. Sequencing of the positive PCR was performed using a BigDye terminator cycle sequencing kit (Applied Biosystems, Foster City, CA), followed by sequence analysis to determine the variant of the detected genes by blasting against the NCBI database.

## 3 Results

**FIGURE 1:**
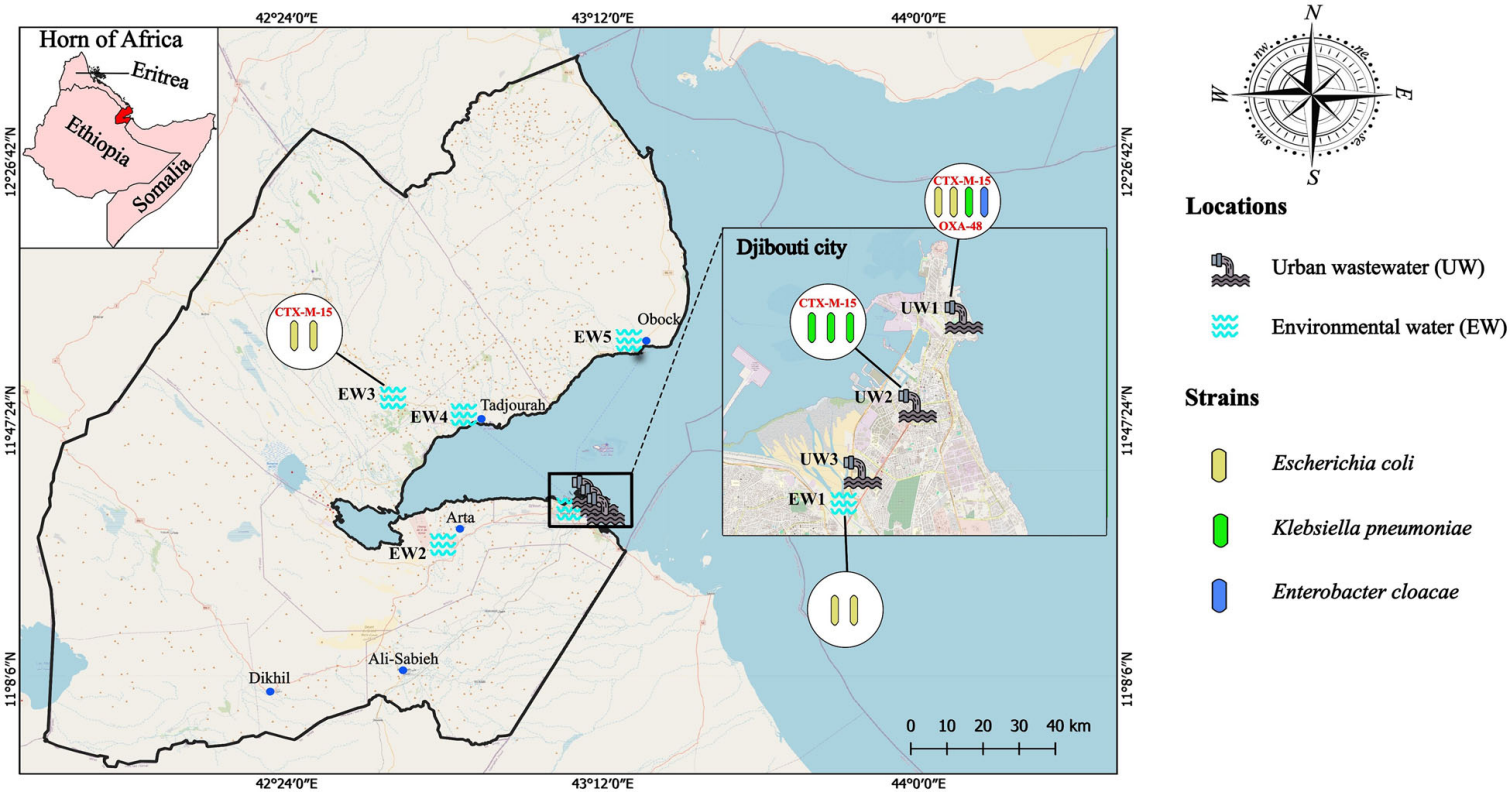
Mapping and bacterial counts of wastewater and environmental water sampling points.

Between February 20 and 28, 2020, a total of 25 wastewater samples were collected across eight distinct locations within the Republic of Djibouti. As shown in Figure 1, these locations include three separate zones of untreated UW in close proximity to residential areas in Djibouti City (population: 1 million inhabitants) and five different EW areas. Of the total samples collected from the eight investigated areas, we obtained bacterial growth in two out of the three selected UW areas and in two out of the five selected EW areas. The bacterial identification by MALDI‐TOF revealed a total of 11 GNB Enterobacteriaceae isolates, including 6 *Escherichia coli* isolates representing the most prevalent species, followed by *Klebsiella pneumoniae* (*n* = 4) and 1 *Enterobacter cloacae* isolate. Seven of these 11 isolates were from UW samples, including hospital wastewater. Indeed, the selective bacterial culture of four hospital wastewater samples on MacConkey medium + ertapenem (0.5 µg/mL) allowed the isolation of two *E. coli* isolates, one *K. pneumoniae* isolate, and one *E. cloacae* isolate from the UW1 zone (Figure 1). However, the bacterial culture of the six UW samples from the UW2 zone on MacConkey medium + cefotaxime (1 µg/mL) allowed the isolation of three *K. pneumoniae* isolates (Figure 1).

For environmental samples, although no bacterial isolates were obtained from the nine samples from areas EW2, EW4, and EW5, four positive cultures were obtained from six environmental wastewater samples from areas EW1 and EW3 (Figure 1). Unlike UW samples, only bacterial culture on MacConkey selective medium + cefotaxime (1 µg/mL) gave a positive result, suggesting the presence of ESBL‐producing isolates. Indeed, the MALDI‐TOF identification of these four positive bacterial cultures revealed the presence of four *E. coli* isolates, including two from area EW1 and two from area EW3 (Figure 1). As presented in Table 1, the antibiotic susceptibility testing revealed multidrug resistance phenotypes for all isolates, with resistance to almost all β‐lactam antibiotics. Indeed, 9 out of the 11 isolates were fully resistant to amoxicillin, amoxicillin/clavulanic acid, cefepime, cefalotin, and ceftriaxone (Table 1). Interestingly, bacterial isolates from hospital wastewater samples exhibited higher resistance levels to the tested antibiotics and were resistant to carbapenems such as Ertapenem (Table 1). Three antibiotics, including imipenem, amikacin, and colistin, remained active against all isolates in this study. Moreover, the global resistance phenotype was similar between isolates from hospital wastewater samples, suggestive of shared resistance genes between these pathogens. According to the AST results, the molecular investigation of resistance genes, especially β‐lactamase genes, by standard PCR and sequencing revealed the presence of class A β‐lactamase in all isolates (Table 1). Surprisingly, we identified the pandemic ESBL *bla*_CTX‐M‐15_ gene in isolates from both urban and environmental wastewater samples (Table 1), suggesting a potential dissemination of resistance genes from hospital settings to EWs. Thus, the *bla_CTX‐M_* and *bla_TEM_* genes were detected in 9 of the 11 isolates (81.8%), and *bla*_SHV_ was detected in three *K. pneumoniae* isolates (27.3%) from UW2 (Table 1). The carbapenemase *bla*_OXA‐48_ gene, conferring specific ertapenem resistance, was detected in four isolates, all from hospital wastewater samples, including *E. coli*, *K. pneumoniae*, and *E. cloacae* isolates (Table 1).

**TABLE 1:** Phenotypic and genotypic resistance profile among gram‐negative bacterial isolates.

Sources	Isolates	AMX	AMC	FEP	TPZ	KF	CRO	ETP	IPM	FF	F	SXT	AK	CIP	DO	CT	CN	β‐Lactamase genes detected
**Hospital wastewater (UW1)**	*Escherichia coli*	**R**	**R**	**R**	**R**	**R**	**R**	**R**	**S**	**S**	**R**	**R**	**S**	**R**	**R**	**S**	**S**	*bla*_CTX‐M‐15_, *bla*_TEM‐214_, *bla*_OXA‐181_
	*E. coli*	**R**	**R**	**R**	**R**	**R**	**R**	**R**	**S**	**S**	**R**	**R**	**S**	**R**	**R**	**S**	**R**	*bla*_CTX‐M‐15_, *bla*_TEM‐214_, *bla*_OXA‐48_
	*Klebsiella pneumoniae*	**R**	**R**	**R**	**R**	**R**	**R**	**R**	**S**	**S**	**R**	**R**	**S**	**R**	**R**	**S**	**R**	*bla*_CTX‐M‐15_, *bla*_TEM‐214_, *bla*_OXA‐48_
	*Enterobacter cloacae*	**R**	**R**	**R**	**S**	**R**	**R**	**R**	**S**	**S**	**S**	**R**	**S**	**R**	**S**	**S**	**R**	*bla*_CTX‐M‐15_, *bla*_TEM‐214_, *bl_a_*_OXA‐48_
**Urban wastewater (UW2)**	*K. pneumoniae*	**R**	**R**	**R**	**S**	**R**	**R**	**S**	**S**	**S**	**S**	**R**	**S**	**S**	**R**	**S**	**S**	*bla*_CTX‐M‐15_, *bla*_TEM‐214_, *bla*_SHV‐82_
	*K. pneumoniae*	**R**	**R**	**R**	**S**	**R**	**R**	**S**	**S**	**R**	**S**	**R**	**S**	**S**	**R**	**S**	**S**	*bla*_CTX‐M‐15_, *bla*_TEM‐214_, *bla*_SHV‐168_
	*K. pneumoniae*	**R**	**R**	**R**	**S**	**R**	**R**	**S**	**S**	**R**	**S**	**R**	**S**	**R**	**R**	**S**	**S**	*bla*_CTX‐M‐15_, *bla*_TEM‐206_, *bla*_SHV‐186_
**Environmental water (EW1)**	*E. coli*	**R**	**R**	**S**	**S**	**R**	**S**	**S**	**S**	**S**	**S**	**R**	**S**	**S**	**R**	**S**	**S**	*bla*_TEM‐141_
	*E. coli*	**R**	**R**	**S**	**S**	**R**	**S**	**S**	**S**	**S**	**S**	**S**	**S**	**S**	**R**	**S**	**S**	*bla*_TEM‐104_
**Environmental Water (EW3)**	*E. coli*	**R**	**R**	**R**	**S**	**R**	**R**	**S**	**S**	**R**	**S**	**S**	**S**	**S**	**R**	**S**	**S**	*bla*_CTX‐M‐15_, *bla*_TEM‐104_
	*E. coli*	**R**	**R**	**R**	**S**	**R**	**R**	**S**	**S**	**S**	**S**	**S**	**S**	**S**	**S**	**S**	**S**	*bla*_CTX‐M‐15_

Abbreviations: AK, amikacin; AMC, amoxicillin/clavulanic acid; AMX, amoxicillin; CIP, ciprofloxacine; CN, gentamicin; CRO, ceftriaxone; CT, colistin; DO, doxycycline; ETP, ertapenem; F, nitrofurantoin; FEP, cefepime; FF, fosfomycin; IPM, imipenem; KF, cefalotin; R, resistance; S, sensitive; SXT, trimethoprim/sulfamethoxazole; TPZ, piperacillin + tazobactam.

## 4 Discussion

Aquatic environments act as important reservoirs of antibiotic resistance determinants and can serve as a route for the dissemination of MDR bacteria into humans and animals [20]. Although the presence of AMR microorganisms in the environment is of global importance, in Africa it is even more critical due to the lack or weakness of antimicrobial use surveillance systems, the unregulated waste disposal processes, and the poor sanitation infrastructures [21, 22, 23, 24]. In this study, we investigated AMR in wastewater samples from collection centers in the City of Djibouti, the capital and largest city of the Republic of Djibouti. This city has the highest human density in the country, with two‐thirds of the total population, and direct discharge of untreated domestic and hospital wastewater into surface water is common there. To the best of our knowledge, this is the first study carried out in Djibouti describing the presence of MDR bacteria in untreated environmental and UW. The identified pathogenic bacteria were *E. coli*, *K. pneumoniae*, and *E. cloacae*. Due to its resilience and ability to exchange resistance genes via horizontal transfer, *E. coli* is frequently recovered from environmental samples [25, 26]. The presence of *E. coli* and *Klebsiella* spp. (which are part of the microbiota of both healthy and sick individuals) in the environment and UW indicates fecal contamination and may increase risks to human health. Other studies in East Africa, including Ethiopia [27, 28], Kenya [29], and Tanzania [30], have also found bacteria in wastewater and EWs. Moreover, MDR bacteria have been found in environmental wastewater from different countries worldwide, including both developed and developing countries (e.g., Switzerland [31], Sweden [32], Australia [33], Brazil [34], Mexico [35], India [36], and Lebanon [37]).

Despite our limited sampling and few results, it is notable that 9 out of the 11 identified isolates were ESBL producers carrying the pandemic *bla*_CTX‐M‐15_ gene, demonstrating its high prevalence in this first study from Djibouti. Studies conducted in Nigeria, Tunisia, and South Africa have also reported high prevalence rates of MDR bacteria in urban and EWs [38, 39, 40]. It is also interesting to note that we detected the carbapenemase enzyme OXA‐48 only in isolates from hospital wastewater. Similar results were reported in Africa in Burkina Faso [41], Congo [42], and South Africa [43], and in different parts of the world [11, 44, 45, 46, 47]. The presence of OXA‐48 carbapenemase in diverse bacterial species within hospital wastewater likely reflects its higher prevalence among hospitalized patients compared to healthy community carriers [48]. This is consistent with a recent study conducted in Djibouti on human isolates, which revealed the presence of strains carrying carbapenemase genes such as OXA‐48, OXA‐23, and different variants of NDM enzymes [49]. Hospitals are a crucial focus for the emergence and spread of AMR, as there is widespread use of antibiotics in hospital settings, contributing to the increase in antibiotic residues and AMR in hospital waste and sewage treatment plants. In recent years, drought in Djibouti and a large population of Ethiopian origin moving within the country have increased the risk of waterborne infectious diseases and affected water access [50, 51]. Therefore, the existence of strains carrying β‐lactamase and carbapenemase genes in Djibouti is not surprising. Although β‐lactams are widely used in the treatment of patients in healthcare facilities in Djibouti, the spread of hospital strains through municipal wastewater treatment plants was not confirmed in this study. Given the small sample size in this study, future studies should collect multiple samples from different points in municipal wastewater treatment plants, such as basins before and after treatment and the discharge area of treated water into the environment. Future studies should also employ methodologies such as membrane filtration and whole‐genome sequencing for bacterial identification and carbapenemase gene detection.

In conclusion, for the first time, we have reported here the prevalence of genes conferring resistance to beta‐lactams and carbapenems in GNB in urban and environmental wastewater in the Republic of Djibouti. Furthermore, these results could serve as a basis for future long‐term surveillance studies and the assessment of trends in infections caused by MDR‐GNB in the Djiboutian population. However, it is necessary to highlight the limitations of our work, related to the number and frequency of samples. Therefore, future studies should be conducted with adequate resources and policies to comprehensively survey antibiotic resistance in the Republic of Djibouti, accounting for spatiotemporal variations.

## Author Contributions

**Ayan Ali Ragueh**: methodology, formal analysis, writing original draft preparation, writing review and editing, funding acquisition. **Ibrahim S. Abdallah**: methodology. **Rachid M. Mouhoumed**: formal analysis. **Mohamed H. Aboubaker**: supervision, funding acquisition. **Seydina M. Diene**: conceptualization, writing original draft preparation, writing review and editing, supervision. All authors have read and agree with this version of the manuscript.

## Ethics Statement

This work has been approved by the national ethics committee of the ministry of environment of Djibouti under the number: 242/DE/2023.

## Consent

The authors have nothing to report.

## Conflicts of Interest

The authors declare no conflicts of interest.

## Supporting information



Supporting Information

## Data Availability

The data that support the findings of this study are available from the corresponding author upon reasonable request.
