# Supplementary material for: Environmental Dissemination of Multidrug‐Resistant Gram‐Negative Pathogens in Djibouti's Wastewaters
Source: Public Health Chall. 2025 Oct 4;4(4):e70139. doi: 10.1002/puh2.70139 (PMC12495887; doi:10.1002/puh2.70139)
Supplement: Supplementary file 1 — Supporting Information [file PUH2-4-e70139-s001.doc]

**Suppl. Table S1** : Primers used in real-time PCR and standard PCR

| **PCR type** | **Primer** | **Sequence (59–39)a** | **Reference** |
| --- | --- | --- | --- |
| ***Real-time PCR*** | CTX-M-a | F: CGGGCRATGGCGCARAC | 1 |
|  |  | R: TGCRCCGGTSGTATTGCC |  |
|  |  | P: CCARCGGGCGCAGYTGGTGAC |  |
|  | CTX-M-B | F: ACCGAGCCSACGCTCAA |  |
|  |  | R: CCGCTGCCGGTTTTATC |  |
|  |  | P: CCCGCGYGATACCACCACGC |  |
|  | TEM | F: TTCTGCTATGTGGTGCGGTA |  |
|  |  | R: GTCCTCCGATCGTTGTCAGA |  |
|  |  | P: AACTCGGTCGCCGCATACACTATTCTCAGA |  |
|  | SHV | F: TCCCATGATGAGCACCTTTAAA |  |
|  |  | R: TCCTGCTGGCGATAGTGGAT |  |
|  |  | P: TGCCGGTGACGAACAGCTGGAG |  |
|  | VIM | F: CACAGYGGCMCTTCTCGCGGAGA | 2 |
|  |  | R: GCGTACGTYGCCACYCCAGCC |  |
|  |  | P:6FAMAGTCTCCACGCACTTTCATGACGACCGCGTCGGCG-TAMRA |  |
|  | NDM | F: GCGCAACACAGCCTGACTTT | 3 |
|  |  | R: CAGCCACCAAAAGCGATGTC |  |
|  |  | P: 6-FAM-CAACCGCGCCCAACTTTGGC-TAMRA |  |
|  | KPC | F: GATACCACGTTCCGTCTGGA | 4 |
|  |  | R: GGTCGTGTTTCCCTTTAGCC |  |
|  |  | P: 6-FAM-CGCGCGCCGTGACGGAAAGC-TAMRA |  |
|  | OXA-23 | F: TGCTCTAAGCCGCGCAAATA | 5 |
|  |  | R: TGACCTTTTCTCGCCCTTCC |  |
|  |  | P: FAM-GCCCTGATCGGATTGGAGAACCA-TAMRA |  |
|  | OXA-24 | F: CAAATGAGATTTTCAAATGGGATGG |  |
|  |  | R: TCCGTCTTGCAAGCTCTTGAT |  |
|  |  | P: FAM-GGTGAGGCAATGGCATTGTCAGCA-TAMRA |  |
|  | OXA-58 | F: CGCAGAGGGGAGAATCGTCT |  |
|  |  | R: TTGCCCATCTGCCTTTTCAA |  |
|  |  | P: FAM-GGGGAATGGCTGTAGACCCGC-TAMRA |  |
|  | OXA-48 | F: TCTTAAACGGGCGAACCAAG | 2 |
|  |  | R: GCGTCTGTCCATCCCACTTA |  |
|  |  | P: 6-FAM-AGCTTGATCGCCCTCGATTTGG-TAMRA |  |
|  | mcr-1 | F: GCAGCATACTTCTGTGTGGTAC | 6 |
|  |  | R: ACAAAGCCGAGATTGTCCGCG |  |
|  |  | P: FAM-GACCGCGACCGCCAATCTTACC-TAMRA |  |
|  | mcr-2 | F: CTGTGCCGTGTATGTTCAGC | 7 |
|  |  | R: TTATCCATCACGCCTTTTGAG |  |
|  |  | P: VIC-TGACCGCTTGGGTGTGGGTA-TAMRA |  |
|  | mcr-3 | F: TGAATCACTGGGAGCATTAGGGC |  |
|  |  | R: TGCTGCAAACACGCCATATCAAC |  |
|  |  | P: FAM-TGCACCGGATGATCAGACCCGT-TAMRA |  |
|  | mcr-4 | F: GCCAACCAATGCTCATACCCAAAA |  |
|  |  | R: CCGCCCCATTCGTGAAAACATAC |  |
|  |  | P: FAM-GCCACGGCGGTGTCTCTACCC-TAMRA |  |
|  | mcr-5 | F: TATCCCGCAAGCTACCGACGC |  |
|  |  | R: ACGGGCAAGCACATGATCGGT |  |
|  |  | P: FAM-TGCGACACCACCGATCTGGCCA-TAMRA |  |
|  | mcr-8 | F: TCCGGGATGCGTGACGTTGC | 8 |
|  |  | R: TGCTGCGCGAATGAAGACGA |  |
|  |  | P: FAM-TCATGGAGAATCGCTGGGGGAAAGC-TAMRA |  |
| ***Standard PCR*** | CTX-M-1 | F: CCCATGGTTAAAAAATCACTGC | 9 |
|  |  | R: CAGCGCTTTTGCCGTCTAAG |  |
|  | CTX-M-9 | F: GCGCATGGTGACAAAGAGAGTGCAA |  |
|  |  | R: GTTACAGCCCTTCGGCGATGATTC |  |
|  | TEM | F: ATGAGTATTCAACATTTCCGTG | 7 |
|  |  | R: TTACCAATGCTTAATCAGTGAG |  |
|  | SHV | F: ATTTGTCGCTTCTTTACTCGC |  |
|  |  | R: TTTATGGCGTTACCTTTGACC |  |
|  | NDM | F: CATTTGCGGGGTTTTTAATG | 3 |
|  |  | R: CTGGGTCGAGGTCAGGATAG |  |
|  | KPC | F: ATGTCACTGTATCGCCGTCT | 4 |
|  |  | R: TTTTCAGAGCCTTACTGCCC |  |
|  | OXA-48 | F: TTGGTGGCATCGATTATCGG | 10 |
|  |  | R: GAGCACTTCTTTTGTGATGGC |  |

aR, reverse; F, forward; P, probe.

**References**

1. Koudokpon H, Dougnon V, Hadjadj L, *et al.* First Sequence Analysis of Genes Mediating Extended-Spectrum Beta-Lactamase (ESBL) bla-TEM, SHV- and CTX-M Production in Isolates of Enterobacteriaceae in Southern Benin. *Int J Infect 2018 54* 2018; **5**: 83194. Available at: https://brieflands.com/articles/iji-83194. Accessed February 28, 2025.

2. Bayssari C Al, Diene SM, Loucif L, *et al.* Emergence of VIM-2 and IMP-15 Carbapenemases and Inactivation of oprD Gene in Carbapenem-Resistant Pseudomonas aeruginosa Clinical Isolates from Lebanon. 2014; **58**: 4966–70.

3. Diene SM, Bruder N, Raoult D, Rolain J-M. Real-time PCR assay allows detection of the New Delhi metallo-β-lactamase (NDM-1)-encoding gene in France. *Int J Antimicrob Agents* 2011; **37**: 544–6.

4. Mathlouthi N, Al-Bayssari C, El Salabi A, *et al.* Carbapenemases and extended-spectrum β-lactamases producing Enterobacteriaceae isolated from Tunisian and Libyan hospitals. *J Infect Dev Ctries* 2016; **10**: 718–27. Available at: https://pubmed.ncbi.nlm.nih.gov/27482803/. Accessed March 24, 2025.

5. Mesli E, Berrazeg M, Drissi M, Bekkhoucha SN, Rolain J-M. Prevalence of carbapenemase-encoding genes including New Delhi metallo-β-lactamase in Acinetobacter species, Algeria. *Int J Infect Dis* 2013; **17**: e739-43.

6. Chabou S, Leangapichart T, Okdah L, Le Page S, Hadjadj L, Rolain J-M. Real-time quantitative PCR assay with Taqman((R)) probe for rapid detection of MCR-1 plasmid-mediated colistin resistance. *New microbes new Infect* 2016; **13**: 71–4.

7. Touati M, Hadjadj L, Berrazeg M, Baron SA, Rolain JM. Emergence of Escherichia coli harbouring mcr-1 and mcr-3 genes in North West Algerian farmlands. *J Glob Antimicrob Resist* 2020; **21**: 132–7.

8. Nabti LZ, Sahli F, Ngaiganam EP, *et al.* Development of real-time PCR assay allowed describing the first clinical Klebsiella pneumoniae isolate harboring plasmid-mediated colistin resistance mcr-8 gene in Algeria. *J Glob Antimicrob Resist* 2020; **20**: 266–71. Available at: https://doi.org/10.1016/j.jgar.2019.08.018.

9. Poirel L, Bonnin RA, Nordmann P. Genetic features of the widespread plasmid coding for the carbapenemase OXA-48. *Antimicrob Agents Chemother* 2012; **56**: 559–62.

10. Poirel L, Bonnin R a, Nordmann P. Genetic features of the widespread plasmid coding for the carbapenemase OXA-48. *Antimicrob Agents Chemother* 2012; **56**: 559–62. Available at: http://www.pubmedcentral.nih.gov/articlerender.fcgi?artid=3256075&tool=pmcentrez&rendertype=abstract. Accessed May 7, 2014.
